# BCAP Regulates Dendritic Cell Maturation Through the Dual-Regulation of NF-κB and PI3K/AKT Signaling During Infection

**DOI:** 10.3389/fimmu.2020.00250

**Published:** 2020-02-18

**Authors:** Yuhui Miao, Ming Jiang, Lu Qi, De Yang, Weihua Xiao, Fang Fang

**Affiliations:** ^1^Department of Medical Oncology, The First Affiliated Hospital of USTC, Division of Life Sciences and Medicine, University of Science and Technology of China, Hefei, China; ^2^Hefei National Laboratory for Physical Sciences at Microscale, The CAS Key Laboratory of Innate Immunity and Chronic Disease, School of Life Sciences, University of Science and Technology of China, Hefei, China; ^3^Institute of Immunology, University of Science and Technology of China, Hefei, China; ^4^Engineering Technology Research Center of Biotechnology Drugs Anhui, University of Science and Technology of China, Hefei, China; ^5^Cancer and Inflammation Program, Frederick National Laboratory for Cancer Research, Center for Cancer Research, National Cancer Institute, Frederick, MD, United States

**Keywords:** dendritic cells, BCAP, Toll-like receptors, *Listeria monocytogenes*, NF-κB signaling, PI3K/AKT signaling

## Abstract

The maturation of dendritic cells (DCs) is essential in adaptive immunity. B cell adapter for phosphoinositide 3-kinase (BCAP) has been shown a divergent activities in cell type dependent manner including B cells, NK cells, macrophages, and plasmacytoid DCs (pDCs), however, its role in conventional DCs (cDCs) remains unknown. Here, we report that BCAP negatively regulates Toll-like receptor-induced cDC maturation and inhibits cDCs from inducing antigen-specific T cell responses, thereby weakening the antibacterial adaptive immune responses of mice in a *Listeria monocytogenes*-infection model. Furthermore, we demonstrate that BCAP simultaneously modulates the activation of the NF-κB and PI3K/AKT signaling by dynamically interacting with, respectively, MyD88 and the p85α subunit of PI3K. Our study thus reveals non-redundant roles for BCAP in regulating cDC maturation and reveals a bilateral signal transduction mechanism.

## Introduction

DCs bridge innate immunity and adaptive immunity through their unique antigen-presenting ability (1, 2). Upon pathogen encounter, DCs undergo a process called “maturation,” characterized by the up-regulation of surface co-stimulatory receptors, major histocompatibility complexes (MHCs), and the production of pro-inflammatory cytokines, all of which are important for the priming of naive T cells (1, 3, 4). A normal maturation of DCs is indispensable for the establishment of powerful innate and adoptive immune responses during infection, and abnormal maturation of DCs is often linked with immune disorders and diseases. While excessive maturation of DCs leads to transplant rejection and the onset of autoimmune disease (5, 6), loss of normal DC maturation results in uncontrollable infection and inflammation. It has become clear that immature DCs mainly elicit immunological tolerance and are critical for the establishment of peripheral tolerance (5, 7, 8), which further underlines the critical role of mature DCs in shaping appropriate adaptive immune responses.

The maturation of DCs is mainly caused by the activation of pattern recognition receptors (9–11), the best-characterized of which are the Toll-like receptors (TLRs). Activation of TLRs induces a series of intracellular signaling cascades including the NF-κB, MAPK, and PI3K/AKT signaling in a MyD88-dependent and/or TRIF-dependent manner (9, 12). TLR-mediated activation of NF-κB and MAPK signaling is responsible for initiating the transcription of pro-inflammatory cytokines and co-stimulatory receptors. The activation of PI3K/AKT signaling in DCs acts mainly as a negative regulator of TLR signaling (13, 14). The activation and crosstalk of these signaling networks are delicately monitored and still require more investigation.

BCAP acts as a signaling adaptor and contains numerous potential protein-protein interaction domains, but does not contain any enzymatic domains (15). Through protein-protein interactions, BCAP can orchestrate multiple intracellular signals downstream of receptor and non-receptor tyrosine kinases. BCAP was initially described as an adaptor for PI3K/AKT signaling transduction downstream of BCR and CD19 in B cells (16, 17). BCAP-activated PI3K/AKT signaling is indispensable for B cell development and activation (18) but inhibits the activation of macrophages and NK cells (19, 20). These paradoxical results suggest that BCAP plays different roles in cell-type specific fashion. In addition to the PI3K/AKT signaling, BCAP also modulates the activation of NF-κB signaling. BCAP-deficient mice have a reduced number of mature B cells due to impacted NF-κB signaling in B cells, suggested a non-redundant role of BCAP in maintaining the NF-κB signaling activation during B cell development (21). However, recent studies demonstrated that BCAP limits the activation of NF-κB signaling by interacting with TIR domain-containing proteins in macrophages (22, 23). Although a recent report demonstrates that BCAP promotes IFN-α production through TLR7/9 signal transduction in pDCs (24), and BCAP has been found to be expressed in cDCs (22), its role in cDCs is yet to be elucidated. These results further highlight the diversity functions of BCAP, as it has the ability to interact with different effector proteins depending on the context.

Here, we describe a role of BCAP as a modulator of cDC maturation and conclude that BCAP acts as a negative regulator of TLR signaling through orchestrating the activation of NF-κB and PI3K/AKT signaling. This dual-regulation properties of BCAP relies on its bilateral interaction with MyD88 and p85α (a subunit of PI3K). Consistently, BCAP impairs TLR-induced cDC maturation, limiting the adoptive immune responses against *Listeria monocytogenes* in mouse model. Collectively, these findings have uncovered a previous unknown strategy for the regulation of cDC maturation in mice.

## Materials and Methods

### Mice

BCAP-deficient mice (*Pik3ap1* knockout mice) on a C57BL/6 background were kindly provided by Tomohiro Kurosaki and characterized as described (18). OT-II mice and CD45.1 mice on a C57BL/6 background were gifts from Zhigang Tian at University of Science and Technology of China. CD11c-DTR mice were obtained from Cai Zhang at Shandong University. All mice were maintained in specific pathogen-free facilities at the University of Science and Technology of China, and all animal experiments were approved by the Ethics Committee of the University of Science and Technology of China.

### Cell Lines

DC2.4 cells were generated as previously described (25) and obtained from Dr. K. L. Rock (Dana-Farber Cancer Institute, Boston, MA). DC2.4 cells were cultured in DMEM (HyClone, SH30021.01) supplemented with 10% fetal bovine serum (Biological Industries USA, 04-001-1 ACS), 25 mM HEPES, 100 IU/ml penicillin (Sangon Biotech, A600135-010), 100 mg/ml streptomycin (Sangon Biotech, SB0494-50g) and 50 μM 2-ME, at 37°C with 5% CO_2_. To induce DC maturation, DC2.4 cells were starved in DMEM containing 1% fetal bovine serum for 4 h, followed by 1 μg/ml of the TLR4 agonist LPS (Sigma-Aldrich LLC, L2880-10MG), 1 μg/ml of the TLR2 agonist pam3csk4 (InvivoGen, PMS-39-02), or 10 μg/ml of the TLR3 agonist poly I:C (InvivoGen, PIW-39-01) stimulation, as indicated.

### Flow Cytometry

The preparation of the cell suspension was performed on an automatic tissue grinder (Miltenyi Biotec) according to manufacturer's instructions. Briefly, mouse spleens were placed in a C-tube with 5 ml PBS, and crushed using the m_spleen_01 program. Single-cell suspensions were washed twice and resuspended in PBS containing 10% rat serum (Future, F001007) at 4°C for 30 min (in order to block Fc receptors), prior to incubation with appropriate antibodies in the dark at 4°C for a further 30 min. The stained cells were subsequently washed and acquired on either the FACS Calibur (BD Bioscience) or the CytoFLEX (Beckman Coulter) flow cytometers. Data were analyzed using FlowJo v10.5 or CytExpert software. The antibodies were listed in Table S1. For the intracellular detection of cytokines, cells were treated with 2.5 ng/ml monensin (Sigma-Aldrich LLC, 22373-78-0) and 20 ng/ml PMA (Sigma-Aldrich LLC, P1585) for 4 h. The Foxp3/Transcription Factor Staining Buffer Set (Invitrogen, 00-5523-00) was used as instructed. The Mouse Inflammatory Cytokines Kit (BD bioscience, 51-9010817) was used for extracellular cytokine detection.

### Plasmids and Transfection

The pLKO.1-shRNA library was purchased from Sigma-Aldrich LLC. For the generation of lentivirus, lentiviral vectors containing an expression cassette of short interfering RNAs were co-transfected with packaging plasmids (VSVG: Ggl: Rev: pLKO.1 =1: 2: 2: 2) into 293T cells. Following a 48 h incubation, supernatants were collected and stored at −80°C until further use. The generation of stable transduced cell lines using lentivirus was performed as previously described (26). The pLKO.1 plasmid, containing scrambled shRNA, was used as a control. Lentivirally-transduced cells were cultured in the presence of 2 μg/ml puromycin (Sangon Biotech, A610593) for 2–3 weeks to achieve the stable expression of the protein of interest.

### Immunoblot Analysis

Cells were harvested and lysed using the radioimmunoprecipitation assay buffer (RIPA buffer, 50 mM Tris-HCl; pH = 7.4, 1% Nonidet P-40, 0.25% sodium deoxycholate, 150 mM NaCl, 1 mM EDTA) containing protease (BBI life science, C600387-0001) and phosphatase inhibitor cocktails (Bimake, B15001). The whole-cell lysate was quantified using the BCA Kit (Pierce, Rockford, 23227), boiled for 10 min with sample loading buffer, loaded onto an SDS-PAGE gel and separated by electrophoresis. Subsequently, proteins were transferred onto a PVDF membrane and incubated with the indicated primary antibodies, followed by HRP-conjugated secondary antibodies. Imaging was performed on the UVITEC Cambridge ALLIANCE4.7 using the ECL Detection kit (Advansta Inc., K12045-D50). The quantification of protein from blots was performed using ImageJ. The antibodies were listed in Table S2.

### Immunoprecipitation Analysis

Cells were stimulated with 1 μg/ml LPS for the indicated time periods and lysed in weak RIPA buffer (0.5% Triton X-100, 120 mM NaCl, 50 mM Tris-HCl; pH = 7.5) containing phosphatase and protease inhibitors. The whole-cell lysate was sequentially incubated with one of the following antibodies: anti-MyD88, anti-p85α, anti-Nck1, anti-BCAP, or anti-pTyr (Santa Cruz, sc-18182) and shaken overnight at 4°C. Protein A/G-agarose beads (MedChemExpress USA, HY-K0202) were added into the mixture and shaken for a further 2 h, prior to elution with 1 × SDS sample buffer. Prepared samples were further analyzed by immunoblot.

### Generation and Purification of Bone Marrow-Derived DCs

The generation of BMDCs was adapted from previous methods (27). Briefly, bone marrow samples from 6 to 8 weeks old WT or BCAP-deficient mice were transferred to RPMI 1640 medium (HyClone, SH0809.01) containing 10% FBS, 20 ng/ml mouse GM-CSF (PEPROTECH Asia, 315-03), and 20 ng/ml mouse IL-4 (PEPROTECH Asia, 214-14). Supernatant cells in culture were abandoned after 2 days, and 70% of the culture medium was replaced with fresh medium every other day. On days 6 and 7, non-adherent and loosely-adherent cells were considered as BMDCs and used for different experiments. In some experiments, 100 ng/ml LPS was added to induce BMDC maturation. BMDCs were further purified (purity > 95%) by positive selection using anti-CD11c microbeads (Miltenyi Biotec, 130-108-338) for *in vivo* transfusion, cytokine production assay and immunoblot analysis. For immunoblot analysis, BMDCs were starved in RPMI 1640 medium containing 1% fetal bovine serum for 4 h before stimulation.

### Real-Time PCR

Total RNA was extracted from BMDCs using the TRIzol reagent (Invitrogen, 15596018) and reverse transcribed using the M-MLV Reverse Transcriptase (Invitrogen, C28025-021) according to the standard manual. The real-time PCR reactions were performed on the ABI7300 real-time PCR system, using the SYBR Green Master Mix (Vazyme, Q111-01). Gene expression results were analyzed by calculating the threshold values (Ct) and fold changes relative to an internal control. The primers were listed in Table S3.

### DC-T Cell Co-culture Experiments

The purification of DCs and T cells was performed by magnetic separation on MS columns (Miltenyi Biotec), using anti-CD11c and anti-CD4 microbeads (both from Miltenyi Biotech, B528411), respectively. Purity (>95%) was assessed by flow cytometry. CD11c^+^ BMDCs were incubated with OVA (500 μg/ml, Sigma-Aldrich LLC, A5503) and LPS (100 ng/ml) for 4 h, then co-cultured with 5 μM CFSE-labeled splenic CD4^+^ T cells from 6 to 8 weeks old OT-II transgenic mice at the indicated ratio for 5 days. T cell proliferation was measured by flow cytometry on the FACS Calibur (BD Bioscience), and analyzed using FlowJo 7.6.1 software. In some experiments, splenic DCs purified with anti-CD11c microbeads from 6 to 8 weeks old WT or BCAP-deficient mice were used instead of BMDCs.

### Antigen Specific T Cell Proliferation Assays

For the generation of CD11c-DTR→ CD45.1 bone marrow chimeras, 6 weeks old CD45.1 mice were lethally irradiated (11Gy) and injected intravenously with 5 × 10^6^ bone marrow cells isolated from CD11c-DTR mice. Recipients were then rested for 6 weeks before use. For detection of antigen specific T cell proliferation, CD11c-DTR→ CD45.1 chimeras were injected intravenously every other day for 5 days with 12 ng DTx (Sigma-Aldrich LLC, D0564) per gram of body weight. 5 × 10^6^ purified WT or BCAP-deficient BMDCs were injected intravenously into chimeras 16 h after the first DTx administration, and 3 × 10^6^ CFSE-labeled CD4^+^ T cell from OT-II mice were subsequently transferred. 500 μg OVA and 100 ng LPS in 20 μl PBS were injected subcutaneously into the hind footpad 1 day post cell transfer. Mice were sacrificed and CD4^+^CFSE^+^ T cell proliferation in popliteal lymph nodes was analyzed by flow cytometry. In some experiment, DTx administration was performed every other day and CFSE-labeled CD4^+^ T cells from OT-II mice were transferred 1 day after the first DTx injection. Subsequently, 3 × 10^6^ WT or BCAP-deficient BMDCs, pre-treated with OVA and LPS for 24 h, were injected subcutaneously into the hind footpad 1 day after cell transfer. Proliferation of specific T cell populations was analyzed as above.

### Bacterial Culture and Infection

The LM-OVA was a generous gift from Jianhua Li (Fudan University) and was cultured as previously described (28). 6 to 8 weeks old CD45.1 mice were lethally irradiated (11Gy) and injected intravenously with 5 × 10^6^ bone marrow cells mixed from CD11c-DTR mice and WT or BCAP-deficient mice at the radio of 1:1. After 6 weeks, the chimeras were injected intravenously every other day with 12 ng DTx per gram of body weight for two times. 1 × 10^5^ CFU of LM-OVA were injected intravenously 1 day after the first DTx injection, and bacteria burden and immune responses in the spleen and liver were analyzed after 3 days. To evaluate antigen specific T cell proliferation, 6 to 8 weeks old WT or BCAP-deficient mice were injected intravenously with 5 × 10^6^ CFSE-labeled CD4^+^ T cell, derived from OT-II mice. 1 × 10^5^ CFU of LM-OVA were injected intravenously 1 day after T cell transfer. CD4^+^CFSE^+^ T cell proliferation in the spleen and liver were analyzed 60 h later. For *Escherichia coli* DH5α (Sangon Biotech, B528411) infection, WT or BCAP-deficient mice were injected intravenously with 5 × 10^6^ bacteria, and the maturation of splenic cDCs was analyzed 12 h later.

### Statistical Analysis

Statistical analyses were performed in GraphPad Prism 6.0 software (GraphPad). The Mann-Whitney tests were used for comparing two sample groups. Data are shown as mean ± SEM. A *p* < 0.05 was considered as a measure of statistical significance. ^*^P < 0.05; ^**^P < 0.01; ^***^P < 0.001; ^****^P < 0.001.

## Results

### BCAP Deficiency in cDCs Exhibited Reduced Susceptibility to *Listeria monocytogenes*

**Figure 1:**
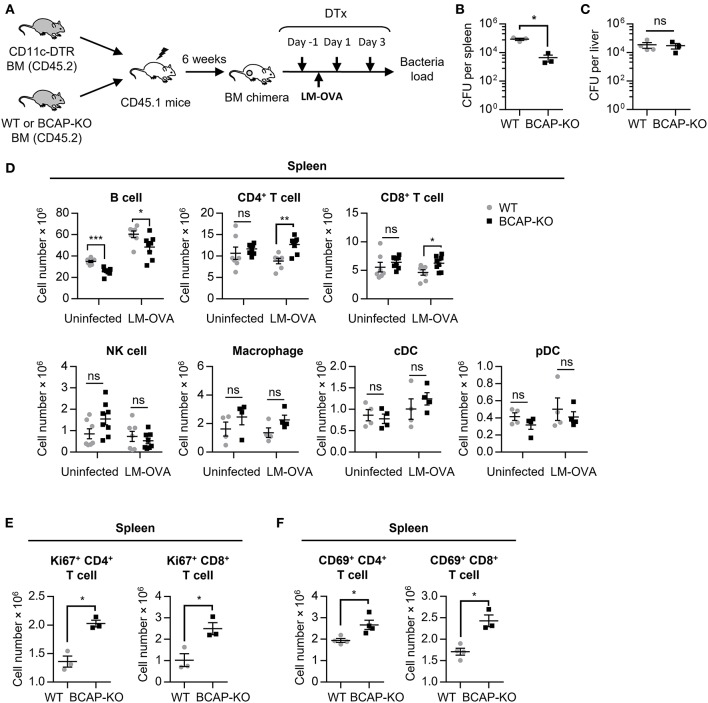
BCAP-deficient cDCs exert strong antimicrobial effects in a mouse model. **(A–F)** Schematic flow chart of the generation of mixed bone marrow chimeras and the LM-OVA infection model **(A)**. The bacterial burden in the spleen **(B)** and liver **(C)** was calculated with limiting dilution, *n* = 3–4 spleens or livers in each group. The total cell numbers of the indicated leukocyte subsets were analyzed by flow cytometry **(D)**, *n* = 4–8 spleens or livers in each group. The expression of Ki67 **(E)** and CD69 **(F)** on T cells was measured by flow cytometry, *n* = 3–4 spleens in each group. WT: CD11c-DTR/WT→ CD45.1 chimeras; BCAP-KO: CD11c-DTR/BCAP-KO→ CD45.1 chimeras. Results are representative of two or three independent experiments. Data are represented as mean ± SEM. ^*^P < 0.05, ^**^P < 0.01, ^***^P < 0.001.

To investigate the role(s) of BCAP in cDCs, the DC-specific BCAP-deficient mice model as previously described (29) were generated by mixing bone marrow from wild-type (WT) or BCAP-deficient (BCAP-KO) mice with bone marrow cells from CD11c-DTR mice at 1:1 ratio, and transferred into irradiated (11 Gy) CD45.1 mice (Figure 1A). DTx administration resulted in the clearance of CD11c^+^ cDCs from CD11c-DTR bone marrow rather than pDCs or CD11c^+^ macrophages in these mixed bone marrow chimeras (Figure S1A), and the residual cDCs were mainly derived from WT bone marrow (CD11c-DTR/WT→ CD45.1 chimeras) or BCAP-deficient bone marrow (CD11c-DTR/BCAP-KO→ CD45.1 chimeras). Next, these chimeras were evaluated for their susceptibility to *Listeria monocytogenes* expressing ovalbumin (LM-OVA) via intravenous injection of 1 × 10^5^ CFU LM-OVA 1 day after the first DTx injection. The LM-OVA burden was much lower than CD11c-DTR/WT→ CD45.1 chimeras in the spleens (Figure 1B) rather than in the livers (Figure 1C) of CD11c-DTR/BCAP-KO→ CD45.1 chimeras, suggesting that BCAP deficiency in cDCs resulted in reduced susceptibility to LM-OVA in mice.

To further investigate potential mechanisms underlying the observed increased clearance of LM-OVA in CD11c-DTR/BCAP-KO→ CD45.1 chimeras, we next analyzed the abundance of different immune cell subsets (Figure S1B). The proportion and total cell number of both CD4^+^ and CD8^+^ T cells in the spleen of CD11c-DTR/BCAP-KO→ CD45.1 chimeras were much higher than CD11c-DTR/WT→ CD45.1 chimeras (Figure S1C, Figure 1D), suggesting enhanced adaptive immune responses in the spleens of the chimeras harboring BCAP-deficient cDCs.

Although NK cells and macrophages plays important roles in host defenses as major innate immune cells, their proportion and cell number had no apparent difference between these chimeric mice after LM-OVA infection (Figure S1C, Figure 1D). These results highlighted the strong impacts of T cells in the observed LM-OVA clearance in these chimeric mice. The proportion and cell number of both CD4^+^ and CD8^+^ T cells in the liver showed no difference between CD11c-DTR/WT→ CD45.1 chimeras and CD11c-DTR/BCAP-KO→ CD45.1 chimeras (Figures S1D,E). These results are in line with the indistinguishable clearance capacities for LM-OVA that we observed for the livers of both groups of mice (Figure 1C), and consistent with the restricted adaptive immune responses in liver (30, 31).

The expression of Ki67 on CD4^+^ and CD8^+^ T cells from the spleens of CD11c-DTR/BCAP-KO→ CD45.1 chimeras was significantly increased compared to spleens of CD11c-DTR/WT→ CD45.1 chimeras (Figure 1E), suggesting increased proliferation of both CD4 and CD8 T cells. Although the proportion of CD69^+^CD4^+^ and CD69^+^CD8^+^ T cells exhibited no difference in the spleens of both kinds of chimeras (Figure S1F), the total cell number of CD69^+^CD4^+^ and CD69^+^CD8^+^ T cells in the CD11c-DTR/BCAP-KO→ CD45.1 chimeras were substantially higher than that of the CD11c-DTR/WT→ CD45.1 chimeras (Figure 1F), in line with the increased clearance of LM-OVA in their spleens.

Together, these results demonstrate that cDC-specific BCAP-deficiency in CD11c-DTR/BCAP-KO→ CD45.1 chimeras exhibited reduced susceptibility to LM-OVA due to their robust T cell responses.

### BCAP-Deficient DCs Exhibit Improved Capacity to Prime T Cells

**Figure 2:**
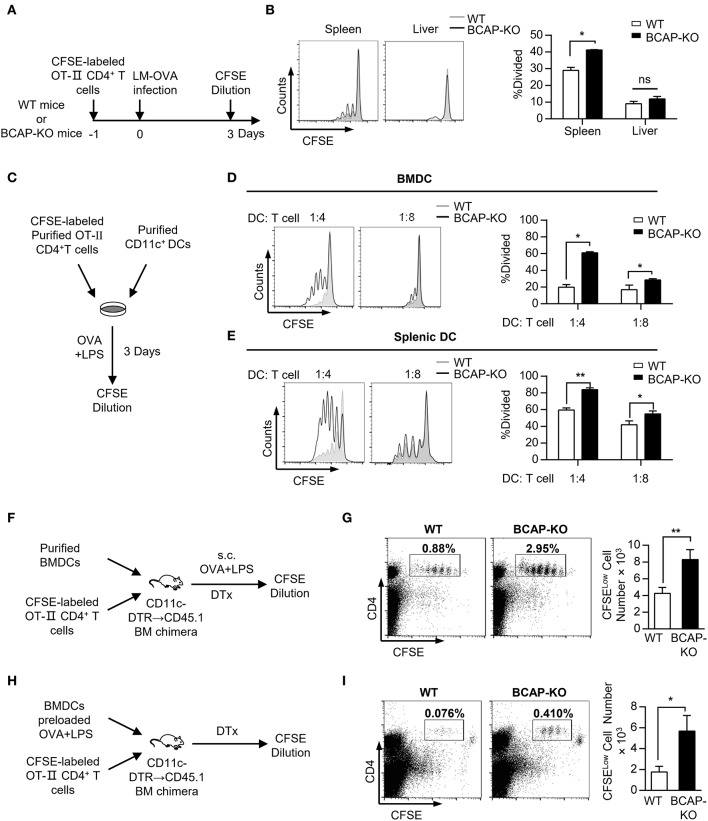
BCAP deficiency increases the potency of DCs in inducing antigen-specific T cell proliferation. **(A,B)** Schematic flow chart of the detection of antigen-specific T cell proliferation during LM-OVA infection in WT or BCAP-deficient mice **(A)**. The proliferation of CFSE^+^CD4^+^ T cells in the spleen and liver was measured by flow cytometry **(B)**, *n* = 3 in each group. **(C–E)** Schematic flow chart of the *in vitro* DC-T cell co-culture experiments **(C)**. T cell proliferation after co-culture with purified BMDCs **(D)** or splenic DCs **(E)** at the indicated ratio for 5 days, evaluated by CFSE dilution, *n* = 3–6 in each group. **(F,G)** Schematic flow chart of T cell proliferation assays with *in vivo* immunization in mice **(F)**. The proportion and total cell number of proliferated CFSE^Low^ T cells were evaluated by CFSE dilution **(G)**, *n* = 6 in each group. **(H,I)** Schema flow chart of T cell proliferation assays with pre-stimulated BMDCs in mice **(H)**. The proportion and total cell number of proliferated CFSE^Low^ T cells were evaluated by CFSE dilution **(I)**, *n* = 8 in each group. CFSE-labeled Viable CD4^+^ T cells were gated before CFSE dilution analysis in **(A–E)**. CD45^+^ cells were gated before CFSE dilution analysis in **(F–I)**. Results are representative of two or three independent experiments. Data are represented as mean ± SEM. ^*^P < 0.05, ^**^P < 0.01.

To address whether BCAP deficiency affects DCs' capacity to prime T cells, we next analyzed the proliferation of antigen-specific T cells during LM-OVA infection. OVA-specific CD4^+^ T cells isolated form OT-II mice exhibited more potent proliferation after transfer into BCAP-deficient mice than after transfer to WT mice during LM-OVA infection (Figures 2A,B), suggesting that BCAP deficiency enhanced the capacity of DCs to prime antigen-specific T cell responses. *Listeria monocytogenes* mainly triggers DC maturation through TLR2 and TLR4 signaling (30–32), thus we next tested whether BCAP affects DC' capacity to prime T cells *in vitro* in the presence of the TLR4 agonist LPS. Similar results were also observed, where bone marrow-derived DCs (BMDCs) from BCAP-deficient mice induced more potent proliferation of CD4^+^ T cells than WT BMDCs, in the presence of OVA and lipopolysaccharide (LPS) (Figures 2C,D). To exclude the effects of macrophages, which may be present in the GM-CSF-induced BMDC population (32), we sorted splenic DCs from WT or BCAP-deficient mice, and observed similar results (Figures 2C,E). Our findings suggest that BCAP deficiency remarkably improves the capacity of DCs to prime T cell responses.

To further address whether BCAP affects the capacity of DCs to prime T cell responses *in vivo*, we utilized another chimera model based on CD11c-DTR mice. Repeated administration of DTx to CD11c-DTR mice is lethal, but this adverse side effect can be overcome by generating CD11c-DTR bone marrow chimeras and transferring them into CD45.1 recipients (33, 34). We thusly generated CD11c-DTR→ CD45.1 chimeras by reconstituting CD45.1 mice with CD11c-DTR bone marrow (Figures S2A,B). DCs in CD11c-DTR→ CD45.1 chimeras could be eliminated by DTx administration (Figure S2C). CD11c-DTR→ CD45.1 chimeras reconstituted with OT-II CD4^+^ T cells and BCAP-deficient BMDCs had increased numbers of OVA-specific CD4^+^ T cells after immunization with OVA and LPS as compared to mice reconstituted with WT BMDCs (Figures 2F,G). To exclude potential interference of residual antigen-presenting cells, we performed alternative experiments in which DTx-treated chimeras were injected together with either WT or BCAP-deficient BMDCs conditioned with a combination of LPS and OVA, following transfusion of OT-II CD4^+^ T cells (Figure 2H). As expected, chimeras reconstituted with pre-activated BCAP-deficient BMDCs also elicited more OVA-specific CD4^+^ T cells than mice reconstructed with pre-activated WT BMDCs (Figure 2I). These results suggested that BCAP restricts the capacity of DCs to prime T cell responses.

### BCAP Deficiency Promotes DC Maturation

**Figure 3:**
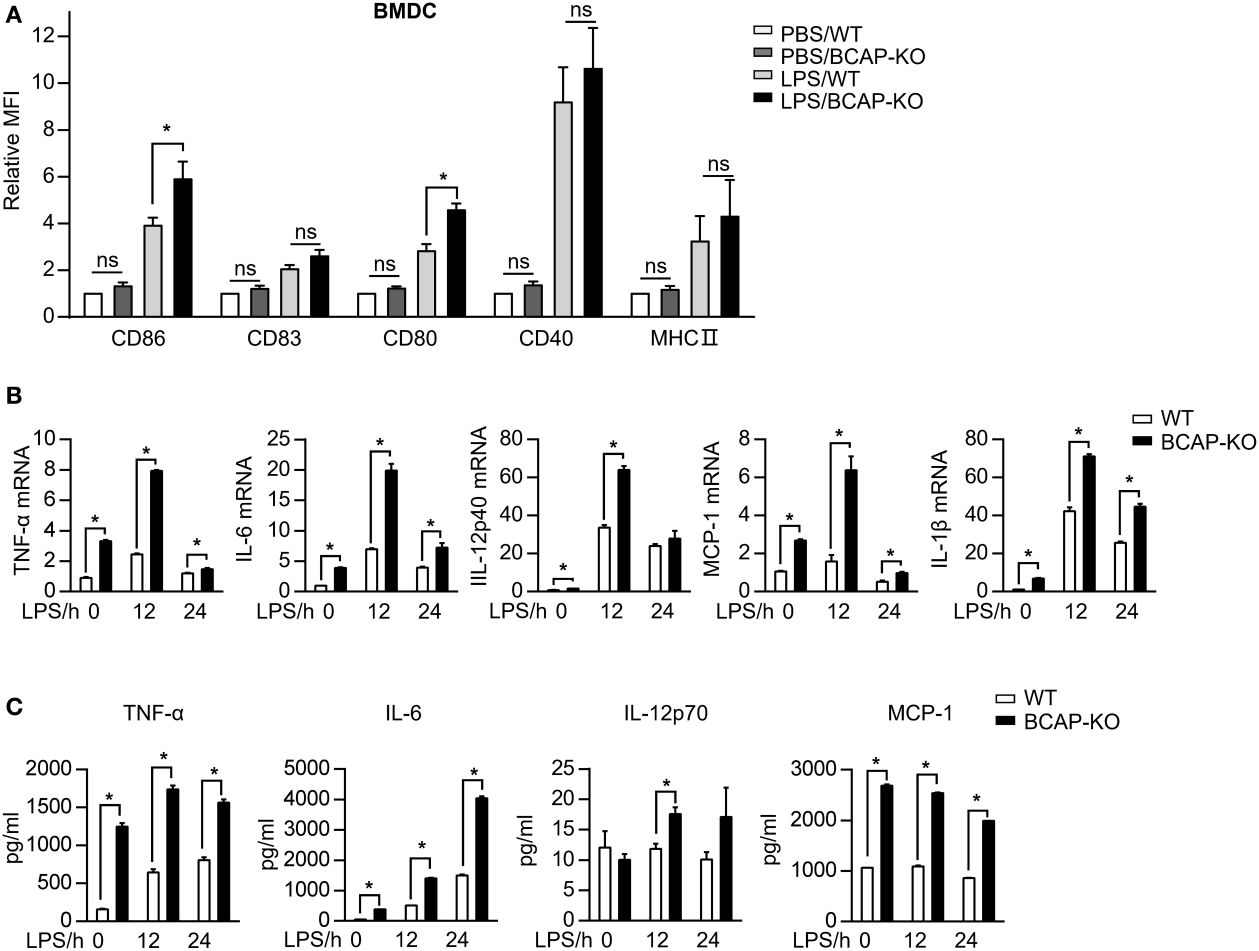
BCAP deficiency promotes LPS-induced DC maturation. **(A)** BMDCs were incubated with 100 ng/ml LPS for 24 h and the expression of selected markers was assessed by flow cytometry, *n* = 5 in each group. **(B,C)** Purified BMDCs were incubated with 100 ng/ml LPS for indicated time points, the mRNA expression levels **(B)** or the production of the indicated cytokines and chemokines **(C)** were measured by qPCR or BD Cytometric Bead Array kit, respectively, *n* = 3 in each group. **(A)** were normalized to WT, and **(B)** to GAPDH. Results are representative of two or three independent experiments. Data are represented as mean ± SEM. ^*^P < 0.05.

The capacity of DCs to prime T cell responses is regulated in different ways, including the expression of MHC class II (MHC II) and co-stimulatory molecules, as well as the production of pro-inflammatory mediators (1, 3). To further investigate the mechanism by which BCAP regulates the capacity of DCs to prime T cell proliferation, we assessed the phenotypic and functional maturation of DCs. The expression of surface markers CD86, CD80, CD40, CD83, as well as MHC class II did not differ between BCAP-deficient and WT immature BMDCs (Figure 3A). In contrast, LPS treatment induced significant upregulated of CD86 and CD80 levels on BCAP-deficient BMDCs compared to WT BMDCs (Figure 3A). Similarly, while steady state levels of CD86 and CD80 on splenic cDCs derived from WT and BCAP-deficient mice were comparable, their expression on splenic cDCs from BCAP-deficient mice were much higher than that from WT mice following infection with *Escherichia coli* DH5α (Figure S3). In addition to phenotypic maturation, BCAP-deficient BMDCs expressed higher *Tnf*, *Il6, Ccl2*, and *Il1b* mRNA levels, compared to WT BMDCs, both following LPS treatment or not (Figure 3B). Consistently, the secretion of TNF-α, IL-6, and MCP-1 proteins by BCAP-deficient BMDCs was also higher, compared to WT BMDCs (Figure 3C).

Together, these results demonstrate that BCAP functions to limits the expression of co-stimulatory molecules and to reduce the production of pro-inflammatory mediators during DC maturation.

### BCAP Regulates Both NF-κB and PI3K/AKT Signaling During TLR-Induced DC Maturation

**Figure 4:**
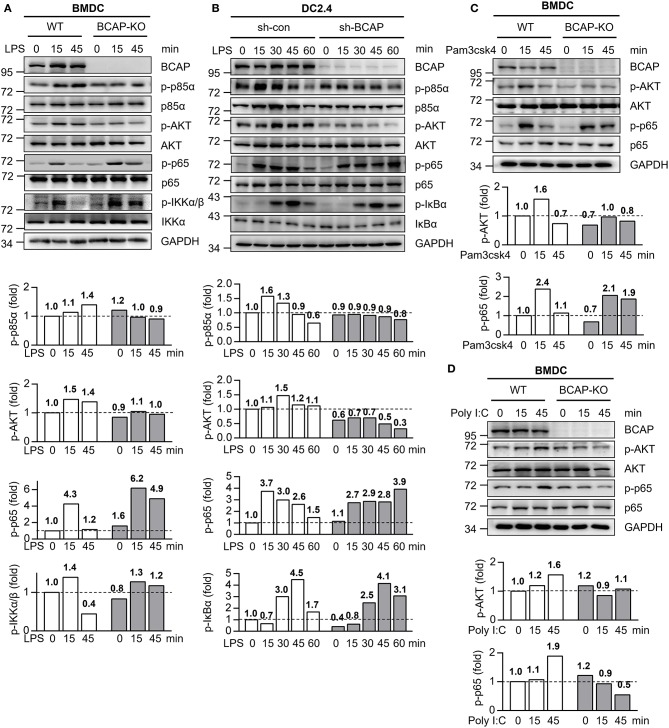
BCAP distinctly regulates different TLR signaling. **(A)** WT BMDCs (WT) and BCAP-deficient BMDCs (BCAP-KO) were stimulated with 100 ng/ml LPS for the indicated time periods, and the phosphorylated (p-) and total proteins in lysates was analyzed by immunoblotting. **(B)** Control DC2.4 cells (sh-con) and BCAP-knockdown DC2.4 cells (sh-BCAP) were stimulated with 1 μg/ml LPS for the indicated time periods, and the phosphorylated (p-) and total proteins in lysates was analyzed by immunoblotting. **(C,D)** WT BMDCs (WT) and BCAP-deficient BMDCs (BCAP-KO) were stimulated with 100 ng/ml pam3csk4 **(C)** or 10 μg/ml poly I:C **(D)** for the indicated time periods, and the phosphorylated (p-) and total proteins in lysates was analyzed by immunoblotting. GAPDH was used as internal control. The quantification of signal intensity is presented as the ratio of phosphorylated protein to internal control, relative to that in unstimulated WT cells, which was set as 1 (dashed line). Results are representative of two or three independent experiments.

To further explore the mechanisms underlying BCAP's modulation of DC function, we next dissected the activation of TLR4 signaling. Following treatment with LPS, BCAP-deficient BMDCs exhibited a reduction in the phosphorylation of p85α subunit of PI3K and AKT, compared to WT BMDCs (Figure 4A), in line with BCAP's established essential role in the activation of PI3K/AKT signaling in several types of immune cells (14, 17, 35). In addition to PI3K/AKT signaling, we also noted that BCAP deficiency affected NF-κB signaling: BCAP-deficient BMDCs exhibited prolonged phosphorylation of IKKα/β and p65 in response to LPS treatment as compared to WT BMDCs (Figure 4A). Similar results were also observed in DC2.4 cells, where BCAP knockdown resulted in attenuated phosphorylation of AKT and p85α but prolonged phosphorylation of IκBα and p65 (Figure 4B).

Similarly, upon TLR2 stimulation with pam3csk4, BCAP deficiency also resulted in declined phosphorylation of AKT and prolonged phosphorylation of p65 in both BMDCs (Figure 4C) and DC2.4 cells (Figure S4A), in line with the similar role of TLR2 and TLR4 in *Listeria* recognition (36–38). However, BCAP deficiency led to reduced phosphorylation of both AKT and p65 during poly I: C induced activation of TLR3 (Figure 4D, Figure S4B), which responsible for the recognition of double-stranded RNA but not LM-OVA (10, 39).

Together, these data suggest that in DCs, BCAP is critical for the initiation of PI3K/AKT signaling, while preventing the excessive activation of NF-κB signaling downstream of TLR4, and thus limited DC maturation.

### BCAP Interacts With MyD88 or p85α to Coordinate TLR4 Signaling

**Figure 5:**
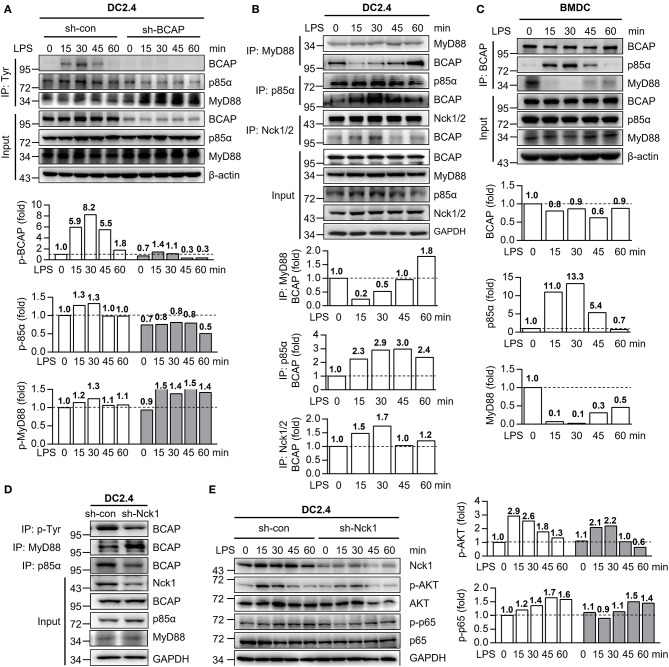
BCAP bilateral interacts with either p85α or MyD88 during DC maturation. **(A)** Control DC2.4 cells and BCAP-knockdown DC2.4 cells were stimulated with 1 μg/ml LPS for the indicated time and lysed, immunoprecipitated with anti-Tyr antibody, and subjected to immunoblotting with anti-MyD88, anti-p85α, and anti-BCAP antibodies. **(B)** DC2.4 cells were stimulated with 1 μg/ml LPS for indicated time periods and lysed, immunoprecipitated with anti-MyD88, anti-p85α or anti-Nck1/2 antibodies, and subjected to immunoblotting with anti-MyD88, anti-p85α, anti-Nck1/2 or anti-BCAP antibodies. **(C)** BMDCs were stimulated with 100 ng/ml LPS for the indicated time periods, lysed, immunoprecipitated with anti-BCAP antibody, and subjected to immunoblotting with anti-MyD88, anti-p85α, or anti-BCAP antibodies. **(D)** Nck1- knockdown DC2.4 cells (sh-Nck1) or control cells were lysed, immunoprecipitated with anti-Tyr, anti-MyD88 or anti-p85α antibodies, and subjected to immunoblotting with anti-BCAP antibody. **(E)** Nck1-knockdown DC2.4 cells or control cells were treated with 1 μg/ml LPS as indicated. The phosphorylation of p65 and AKT were detected using immunoblotting. GAPDH or β-actin were used as internal controls. The quantification of signal intensity is presented as the ratio of phosphorylated protein to internal control, relative to that in unstimulated WT cells, which was set as 1 (dashed line). Input in **A–D** was sampled before immunoprecipitation. Results are representative of two or three independent experiments.

The phosphorylation of BCAP is critical for the initiation of PI3K/AKT signaling in plasmacytoid DCs and TLR-mediated production of cytokines in macrophages (40, 41). To investigate how BCAP modulates both NF-κB and PI3K/AKT signaling in DCs, we measured the phosphorylation of BCAP in DCs in response to LPS stimulation. Interestingly, the phosphorylation of BCAP in DC2.4 cells increased at first, but returned to normal levels within 1 h post LPS treatment (Figure 5A). MyD88 and p85α exhibited similar phosphorylation trends (Figure 5A). Moreover, BCAP knockdown resulted in declined phosphorylation of p85α and prolonged the phosphorylation of MyD88 upon LPS induction (Figure 5A), suggesting that BCAP may somehow orchestrate NF-κB and PI3K/AKT signaling though p85α and MyD88. Consistently, previous studies identified a direct interactions between BCAP and MyD88 during TLR activation in macrophages (22), and between BCAP and p85α during CD19 engagement in B cells (17). Indeed, the interaction between BCAP and MyD88 in LPS-stimulated DC2.4 cells was initially lost and then recovered within 1 h, while the interaction between BCAP and p85α increased initially but became weaker upon (Figure 5B). Similar results were also observed in BMDCs (Figure 5C).

Next, we investigated whether the interaction of BCAP with MyD88 and with p85α affected their phosphorylation. Non-catalytic region of tyrosine kinase (Nck) is an adaptor that connect receptor and non-receptor tyrosine kinases to intracellular signaling networks (42, 43). In B cells, the phosphorylation of BCAP is regulated by the adaptors Nck1 and Nck2 (44, 45). Similarly, the interaction between BCAP and Nck1 became stronger after LPS treatment of DCs (Figure 5B). Moreover, knockdown of Nck1 dramatically reduced the phosphorylation of BCAP, which was accompanied by stronger BCAP–MyD88 interaction and weaker BCAP–p85α interaction (Figure 5D). Knockdown of Nck1 also slightly inhibited both PI3K/AKT and NF-κB signaling (Figure 5E).

Together, these data suggest that the phosphorylation of BCAP controls its interaction with MyD88 and p85α, thus dynamically dual-regulates NF-κB and PI3K/AKT signaling during LPS-induced TLR4 activation in DCs.

## Discussion

**Figure 6:**
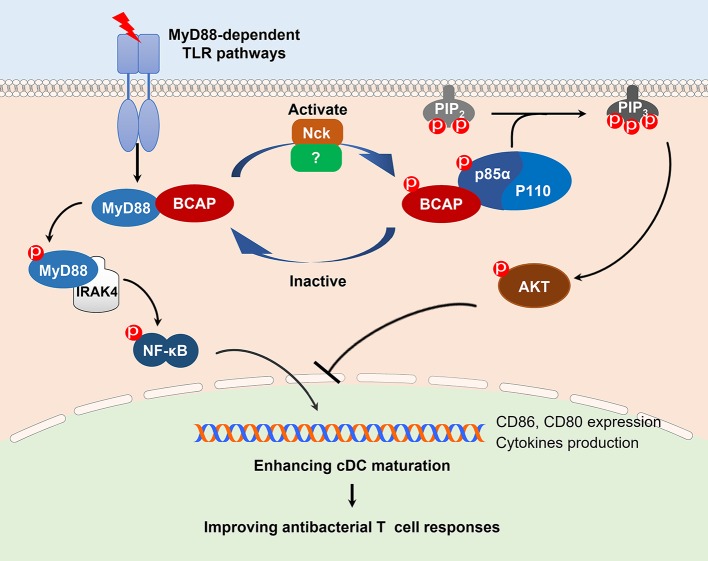
A schematic model of BCAP regulation of NF-κB and PI3K signaling. Upon stimulation with a MyD88-dependent TLR trigger such as LPS, BCAP dissociates from MyD88 and instead interacts with the p85α/p110 complex; collectively, this results in the activation of both the MyD88/NF-κB and PI3K signaling. When BCAP is dephosphorylated, it dissociates from the p85α/p110 complex and re-associates with MyD88, resulting in the inactivation of the both MyD88/NF-κB and PI3K signaling. This bilateral and dual-action regulatory mechanism of BCAP plays a non-redundant role in cDC maturation and antibacterial T cell responses. In addition, Nck mediates the phosphorylation of BCAP during the process.

In this study, we identified a role of BCAP in restricting TLR-induced cDC maturation and modulating the capacity of cDCs to prime T cell responses, through a refined bilateral regulation of NF-κB and PI3K/AKT signaling. Although this kind of dual-regulation by BCAP was speculated previously (14), the detailed mechanism(s) still much unknown. Our results confirmed this speculation and offered more details, where MyD88-dependent TLR activation results in phosphorylation of BCAP, and thus disrupts the BCAP-MyD88 complex. Disengagement from this complex frees MyD88 to activate NF-κB signaling, and phosphorylated BCAP enables to bind the p85α subunit of PI3K, thereby initiating the activation of PI3K/AKT signaling (Figure 6). However, there remains some questions to be investigated, for example, whether other proteins involved in the signal complex, and what triggers the de-phosphorylation of BCAP thereby preventing excessive activation of NF-κB and PI3K/AKT signaling.

A recent report has described a non-redundant role of BCAP in regulating the production of IFN-α of pDCs in mouse systemic lupus erythematosus model (24), which seems contrary to our observations, where BCAP inhibits the production of pro-inflammatory cytokines during the maturation of cDCs. However, the production of IFN-α by activated pDCs is controlled by IRF7 signaling, while the production of inflammatory cytokines by activated pDCs is controlled by NF-κB signaling (46). Thus, these results represent different roles of BCAP in distinct signal transduction processes, and further address the diversity of functions of BCAP in different cell contexts.

The phosphorylation of BCAP is mediated by distinct phosphokinases in different cells. Syk mediated the tyrosine phosphorylation of BCAP during BCR activation in B cells (16). While BCAP is constitutively tyrosine-phosphorylated independent on Syk in macrophages (20). Recent studies also reported that Fyn and Lyn rather than Syk medicated the tyrosine phosphorylation of BCAP in pDCs but not in cDCs (41). The diverse mechanisms of BCAP phosphorylation in different cells points to divergent cell-specific signaling networks, which may account for the diverse functions of BCAP in different cells. Although during TLR-medicated cDC maturation, we also observed a dramatic enhanced phosphorylation of BCAP, but the phosphokinases medicated BCAP phosphorylation in cDCs is still unclear.

BCAP affects the activation of NF-κB signaling though at least three ways. First, BCAP restricts the expression of c-Rel and RelA (p65), therefor down-regulates NF-κB signaling in resting B cells and mature B cells (21). Second, the activation of PI3K/AKT signaling mediated by BCAP limited the expression of NF-κB response genes in macrophages (20, 40). Last but not least, BCAP interacts with MyD88 directly through its TIR domain, limiting the transduction of NF-κB signaling downstream of TLRs in macrophages (22). In our study, BCAP deficiency in DCs results in declined activation of PI3K/AKT signaling, as well as prolonged activation of NF-κB signaling in MyD88-dependent manner within 1 h post TLR4 activation. However, whether BCAP affects the expression of c-Rel and p65 in a longer time periods is still unclear, although their expression was not affected by BCAP during TLR-mediated macrophage activation (35).

Since BCAP deficiency results in the dysregulation of NF-κB and PI3K/AKT signaling downstream of both TLR2 and TLR4, we hypothesize that BCAP acts as a negative regulator downstream of all MyD88-dependent TLRs. To validate this hypothesis, it is necessary to examine the impact of BCAP deficiency on signal transduction upon the activation of different TLRs. Furthermore, detailed functional experiments *in vitro* and *in vivo* are needed to confirm this hypotheses. Although the TLR3 agonist activates the TRIF- rather than the MyD88-mediated signal cascade, BCAP also seems to modulate its signal transduction in a unique manner. This suggests that BCAP may also interacts with TRIF in shaping TLR3 signaling, since it is reported to interact with many TIR domain-containing adaptors besides MyD88, like TRAM and Mal (23, 47). However, detailed mechanisms remain to be studied.

Given that LPS-induced NF-κB signaling transduction is responsible for the up-regulation of CD86 and CD80 (30–32), the enhanced expression of CD86 and CD80 on BCAP-deficient DCs that we observed may results from prolonged activation of NF-κB signaling upon LPS stimulation. Similarly, the increased production of pro-inflammatory mediators by BCAP-deficient DCs may also be attributed to prolonged NF-κB signaling (33–35). Although the production of IL-12 is also controlled by NF-κB (11, 33) and indeed we detected higher levels of *Il12b* mRNA expression in BCAP-deficient BMDCs during LPS treatment, the secretion of IL-12p70 was not substantially different between WT BMDCs and BCAP-deficient BMDCs.

In this study, we designed a DC-specific BCAP-deficient mice model through mixed bone marrow chimeric mice, which were widely used in investigating development and functions of cDCs (29, 48, 49). Considering that BCAP deficiency is known to damage the priming and differentiation of both CD4^+^ T cells and CD8^+^ T cells (15, 50). In our CD11c-DTR/BCAP-KO→ CD45.1 chimera model, which contained 50% BCAP-deficient T cells, the enhanced T cell responses could not be attributed to the BCAP-deficient T cells. Thus, it is reasonable to speculate that BCAP-deficient cDCs might be responsible for the improved T cell responses that observed in our chimeras model during LM-OVA infection.

Our results highlight the importance of BCAP in restricting cDC maturation, which deepens our knowledge of BCAP in the regulation of immune responses, since BCAP works differently in different immune cells. We have also described the detailed mechanisms by which BCAP dynamically modulates distinct signaling, information that will be invaluable for investigating similar questions.

## Data Availability Statement

All datasets generated for this study are included in the article/Supplementary Material.

## Ethics Statement

The animal study was reviewed and approved by the Ethics Committee of the University of Science and Technology of China.

## Author Contributions

WX and DY conceived the project and designed the experiments. FF constructed the study model. FF, YM, MJ, and LQ preformed the experiments and analyzed the data. YM, FF, and MJ wrote the manuscript. All authors reviewed the manuscript.

### Conflict of Interest

The authors declare that the research was conducted in the absence of any commercial or financial relationships that could be construed as a potential conflict of interest.
