# Supplementary material for: BCAP Regulates Dendritic Cell Maturation Through the Dual-Regulation of NF-κB and PI3K/AKT Signaling During Infection
Source: Front Immunol. 2020 Feb 18;11:250. doi: 10.3389/fimmu.2020.00250 (PMC7040100; doi:10.3389/fimmu.2020.00250)
Supplement: Supplementary file 1 [file Data_Sheet_1.pdf]

Supplementary Tables

Supplementary Table 1 Antibodies used in flow cytometry

REAGENT	SOURCE	IDENTIFIER
FITC anti-mouse CD3	Biolegend	Cat#100305
PerCP-Cy5.5 anti-mouse CD8	Biolegend	Cat#100734
APC anti-mouse NK1.1	Biolegend	Cat#108710
PE anti-mouse F4/80	Biolegend	Cat#123110
FITC anti-mouse/human CD11b	Biolegend	Cat#101206
Alexa Fluor® 700 anti-mouse CD19	Biolegend	Cat#115528
PE/Cy7 anti-mouse CD4	Biolegend	Cat#100528
APC/Cyanine7 anti-mouse CD45	Biolegend	Cat#103116
Brilliant Violet 650™ anti-mouse I-A/I-E	Biolegend	Cat#107641
APC/Cyanine7 anti-mouse CD11c	Biolegend	Cat#117324
Brilliant Violet 785™ anti-mouse/human CD45R/B220	Biolegend	Cat#103246
FITC anti-mouse CD86	BD Biosciences	Cat# 553691
PE anti-mouse CD83	BD Biosciences	Cat# 558205
PerCP-Cy5.5 anti-mouse CD80	BD Biosciences	Cat# 560526
APC anti-mouse CD40	BD Biosciences	Cat# 558695
FITC anti-mouse CD11c	BD Biosciences	Cat# 557400
APC anti-mouse CD11c	BD Biosciences	Cat# 550261
PE anti-mouse CD11c	BD Biosciences	Cat# 553802
FITC anti-mouse I-A/I-E	BD Biosciences	Cat# 553623
PE anti-mouse I-A/I-E	BD Biosciences	Cat# 558593
FITC anti-mouse CD4	BD Biosciences	Cat# 553047
APC anti-mouse CD4	BD Biosciences	Cat# 561091
PerCP-Cy5.5 anti-mouse CD45.2	BD Biosciences	Cat# 552950
APC anti-mouse CD45.1	BD Biosciences	Cat# 558701

Supplementary Table 2 Antibodies used in immunoblot analysis

REAGENT	SOURCE	IDENTIFIER
Mouse anti-human/mouse BCAP	R&D Systems	Cat# MAB4857
Rabbit anti-mouse phospho-IKKα/β (Ser176/180)	Cell Signaling Technology	Cat# 2697
Rabbit anti-mouse IKKα	Cell Signaling Technology	Cat# 2682
Rabbit anti-mouse phosphor-p65 (Ser536)	Cell Signaling Technology	Cat# 3033
Mouse anti-mouse p65	Cell Signaling Technology	Cat# 6956
Mouse anti-mouse IκBα	Cell Signaling Technology	Cat# 9247
Rabbit anti-mouse phospho-IκBα(Ser32)	Cell Signaling Technology	Cat# 2859
Rabbit anti-mouse Phospho-PI3 Kinase p85 (Tyr458)/p55 (Tyr199)	Cell Signaling Technology	Cat# 4228
Rabbit anti-mouse phospho-AKT (Ser473)	Cell Signaling Technology	Cat# 4058
Rabbit anti-mouse p44 MAP Kinase (Erk1)	Cell Signaling Technology	Cat# 4372
Rabbit anti-mouse p44/42 MAPK (Erk1/2)	Cell Signaling Technology	Cat# 9102
Mouse anti-mouse Phospho-p44/42 MAPK (Erk1/2) (Thr202/Tyr204)	Cell Signaling Technology	Cat# 9106
Rabbit anti-mouse p38 MAPK	Cell Signaling Technology	Cat# 9212
Rabbit anti-mouse Phospho-p38 MAPK (Thr180/Tyr182)	Cell Signaling Technology	Cat# 9211
Mouse anti-mouse Nck1	Cell Signaling Technology	Cat# 12778
Rabbit anti-mouse Phospho-IRF-3 (Ser396)	Cell Signaling Technology	Cat# 4947
Mouse anti-mouse p85α	Santa Cruz Biotechnology	Cat# sc-1637
Mouse anti-mouse MyD88	Santa Cruz Biotechnology	Cat# sc-74532
Mouse anti-mouse β-actin	Proteintech	Cat# 60008-1
Mouse anti-mouse GAPDH	Proteintech	Cat# 60004-1-Ig
HRP Goat anti-mouse IgG	Biolegend	Cat# 405306
HRP Donkey anti-rabbit IgG	Biolegend	Cat# 406401
Rabbit anti-p-Tyr antibody (PY350)	Santa Cruz Biotechnology	Cat# sc-18182

Supplementary Table 3 Primers used in Real-Time PCR

GENES	SEQUENCE
Tnf	forward: 5'-CCTGTAGCCCACGTCGTAG-3'<br>Reverse:5'-GGGAGTAGACAAGGTACAACCC-3'
Il6	Forward:5'-CCAAGAGGTGAGTGCTTCCC-3'<br>Reverse:5'-CTGTTGTTCAGACTCTCTTCCCT-3'
Il12b	Forward: 5'-TGGTTTGCCATCGTTTTGCTG-3'<br>Reverse:5'-ACAGGTGAGGTTCACTGTTTCT-3'
Il-1b	Forward:5'- GCAACTGTTCCTGAACTCAACT-3'<br>Reverse:5'- ATCTTTTGGGGTCCGTCAACT-3'
Ccl2	Forward:5'-TTAAAAACCTGGATCGGAACCAA-3'<br>Reverse:5'-GCATTAGCTTCAGATTTACGGGT-3'

Supplementary Figures

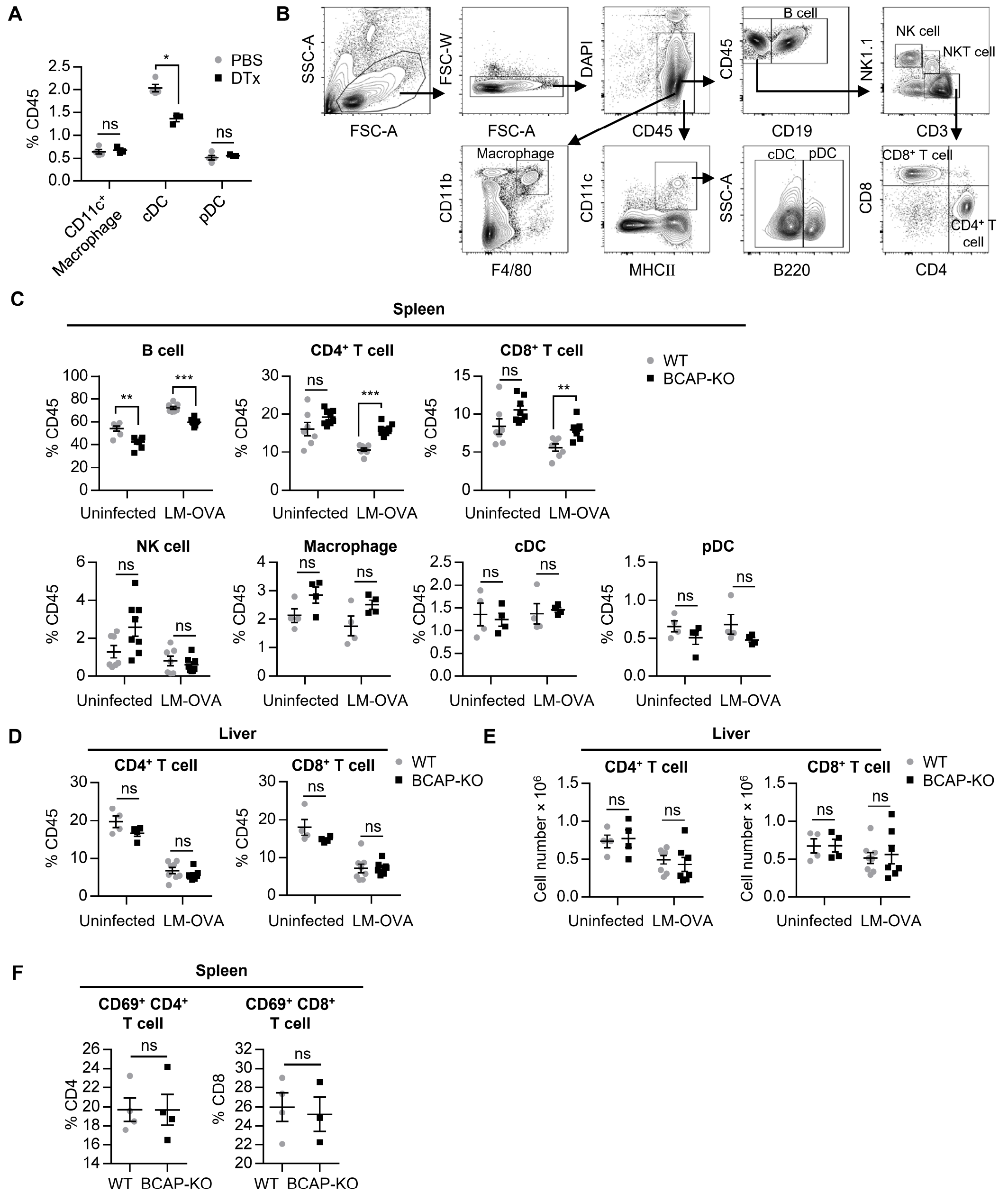

Supplemental Figure 1. BCAP deficiency in cDCs affects antimicrobial immune responses in mice. **A.** CD11c-DTR/WT→CD45.1 chimeras were injected intravenously every other day with 12 ng DTx per gram of body weight for two times, the proportion of indicated cells in the spleen were analyzed 2 days after the last injection using flow cytometry, n=3-4 spleens in each group. **B**. The gating strategy for the characterization of immune cell subsets, by flow cytometry. **C**. the proportion of indicated leukocyte subsets in the spleen of LM-OVA uninfected and infected chimeras. **D-E**. The proportion (**D**) and total cell number (**E**) of each immune cell subsets in the liver of LM-OVA uninfected and infected chimeras. **F.** The expression of CD69 on T cells was measured by flow cytometry, n=3-4 spleens in each group. WT: CD11c-DTR/WT→CD45.1 chimeras; BCAP-KO: CD11c-DTR/BCAP-KO→ CD45.1 chimeras. Data are represented as mean ± SEM, * $P < 0.05$; ** $P < 0.01$; ***

P < 0.001.

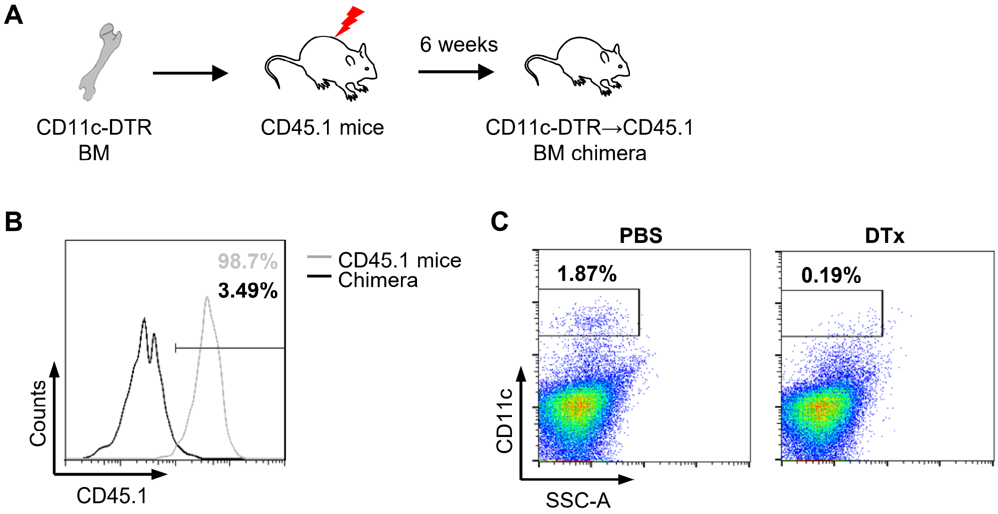

Supplemental Figure 2. The generation of CD11c-DTR→CD45.1 chimeras. A. Schema flow chart of the generation of CD11c-DTR→CD45.1 chimeras. **B.** the expression of CD45.1 on splenic DCs from recipient mice (WT) and CD11c-DTR→CD45.1 chimeras (Chimera) was measured by flow cytometry. **C.** The proportion of DCs in spleen of CD11c-DTR→CD45.1 chimeras treated with 12 ng/g DTx or PBS for the same volume was measured by flow cytometry.

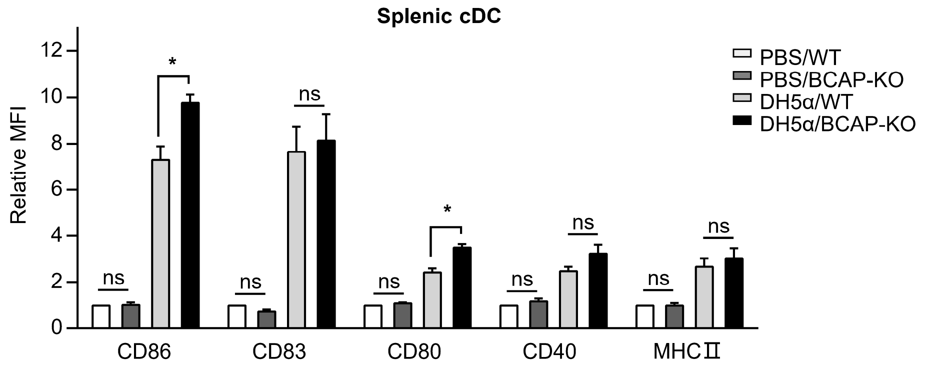

Supplemental Figure 3. The expression of surface markers on splenic DCs after DH5α infection. WT or BCAP-deficient mice were infected via intravenously injection with 5×10^6 CFU *E. coli* DH5α, and the expression of selected molecules by the $MHCII^+$ $CD11c^{Hi}$ DC population in the spleen was measured by flow cytometry 12 hours later, n=3 in each group. Data are represented as mean ± SEM, * $P < 0.05$.

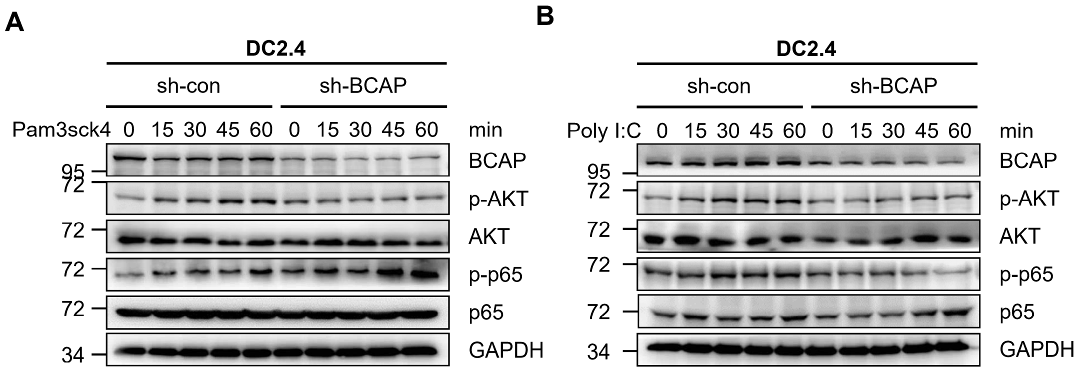

Supplemental Figure 4. Divergent responses to TLR2 and TLR3 signaling in BCAP-knockdown DCs. **A-B.** BCAP-knockdown DC2.4 cells (sh-BCAP) or control cells (sh-con) were stimulated with 100 ng/ml pam3csk4 (**A**) or 10 µg/ml poly I:C (**B**) as indicated. The phosphorylation of AKT and p65 in whole-cell lysates was analyzed by immunoblotting with specific antibodies. GAPDH was used as internal control.
